# Supplementary material for: Identifying and characterizing Stagonosporopsis cucurbitacearum causing spot blight on Pinellia ternata in China
Source: PeerJ. 2022 Apr 13;10:e13278. doi: 10.7717/peerj.13278 (PMC9013236; doi:10.7717/peerj.13278)
Supplement: Supplemental Information 1 [file peerj-10-13278-s001.docx]

Supplemental Table 1 Morphology and pathogenicity of the 15 isolates

| **Name** | **PCR results** | **Morphological character** | **Pathogenicity test** |
| --- | --- | --- | --- |
| AG-1 | *Stagonosporopsis cucurbitacearum* | 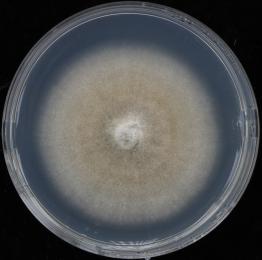 | 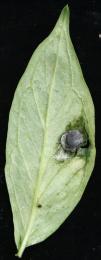 + + + + |
| AG-2 | *Fusarium graminearum* | 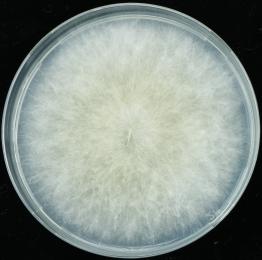 | 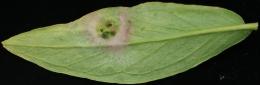 + |
| AG-3 | *Stagonosporopsis cucurbitacearum* | 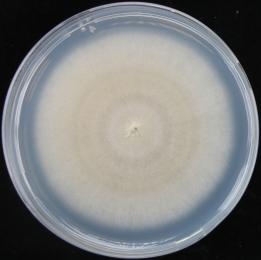 | 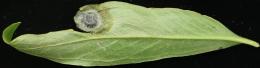 + + + + |
| AG-4 | *Stagonosporopsis cucurbitacearum* | 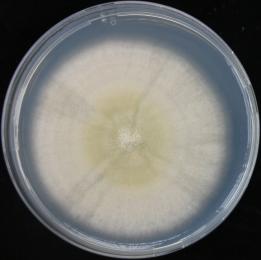 | 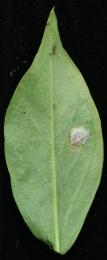 + |
| AG-5 | *Stagonosporopsis cucurbitacearum* | 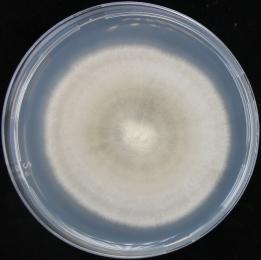 | 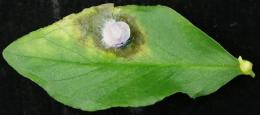 + + + + + |
| AG-6 | *Stagonosporopsis cucurbitacearum* | 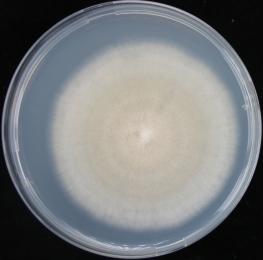 | 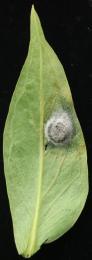 + + + + |
| AG-7 | *Fusarium*  *incarnatum* | 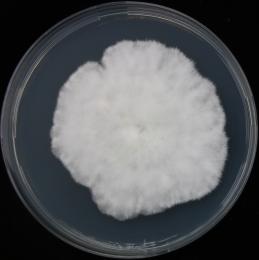 | 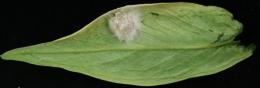 + |
| AG-8 | *Stagonosporopsis cucurbitacearum* | 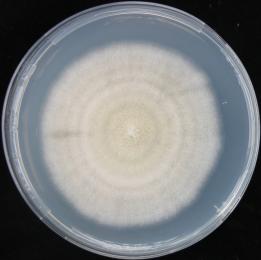 | 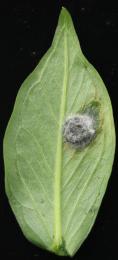 + + + + |
| AG-9 | *Stagonosporopsis cucurbitacearum* | 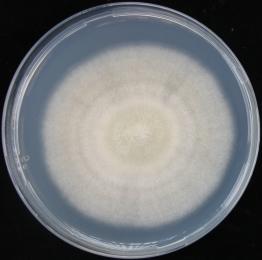 | 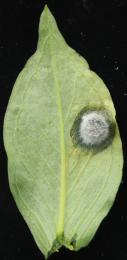 + + + + |
| AG-10 | *Penicillium* sp. | 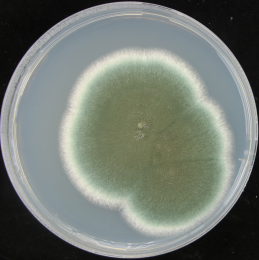 | 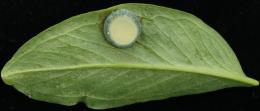 _ |
| AG-11 | *Stagonosporopsis cucurbitacearum* | 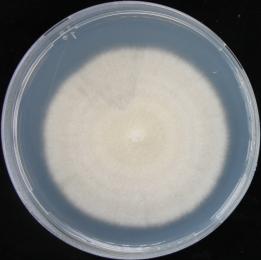 | 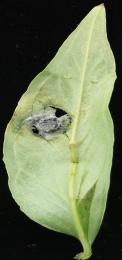 + + + + |
| AG-12 | *Stagonosporopsis cucurbitacearum* | 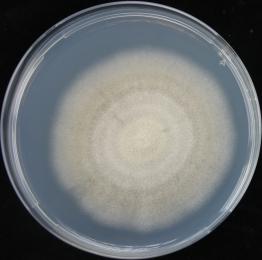 | 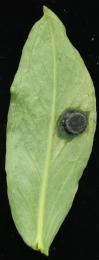 + + + |
| AG-13 | *Stagonosporopsis cucurbitacearum* | 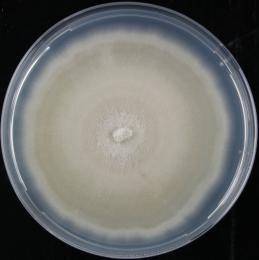 | 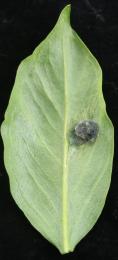 + + |
| AG-14 | *Penicillium* sp. | 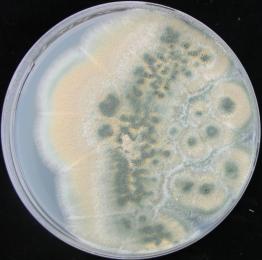 | 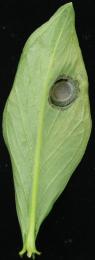 _ |
| AG-15 | *Stagonosporopsis cucurbitacearum* | 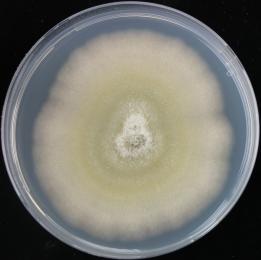 | 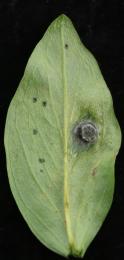 + + + |
